# Sophoridine Suppresses Herpes Simplex Virus Type 1 Infection by Blocking the Activation of Cellular PI3K/Akt and p38 MAPK Pathways

**DOI:** 10.3389/fmicb.2022.872505

**Published:** 2022-06-10

**Authors:** Qiong Tang, Fei Luan, An Yuan, Jiayi Sun, Zhili Rao, Baojun Wang, Yao Liu, Nan Zeng

**Affiliations:** ^1^State Key Laboratory of South Western Chinese Medicine Resources, School of Pharmacy, Chengdu University of Traditional Chinese Medicine, Chengdu, China; ^2^Innovative Institute of Chinese Medicine and Pharmacy, Chengdu University of Traditional Chinese Medicine, Chengdu, China; ^3^School of Laboratory Medicine, Chengdu Medical College, Chengdu, China

**Keywords:** sophoridine, HSV-1, antiviral, PI3K/Akt pathway, p38 MAPK pathway

## Abstract

Herpes simplex virus type 1 (HSV-1) is a ubiquitous and important human pathogen capable of causing significant clinical diseases ranging from skin damage to encephalitis, particularly in immunocompromised and neonatal hosts. Currently, widely used nucleoside analogs, including acyclovir and penciclovir, have some limitations in their use due to side effects and drug resistance. Herein, we report sophoridine's (SRI) dramatic inhibition of HSV-1 replication *in vitro*. SRI exhibited a remarkable inhibitory influence on HSV-1 virus-induced cytopathic effect and plaque formation, as well as on progeny viruses in Vero and HeLa cells, with selection indexes (SI) of 38.96 and 22.62, respectively. Moreover, SRI also considerably suppressed HSV-1 replication by hindering the expression of viral immediate-early (ICP0 and ICP22), early (ICP8 and TK), and late (gB and gD) genes and the expression of viral proteins ICP0, gB, and gD. We suggest that SRI can directly inactivate viral particles and block some stages in the life cycle of HSV-1 after adsorption. Further experiments showed that SRI downregulated the cellular PI3K/Akt signaling pathway and obstructed HSV-1 replication even more. Most importantly, SRI markedly repressed HSV-1-induced p38 MAPK pathway activation. Collectively, this natural bioactive alkaloid could be a promising therapeutic candidate against HSV-1 *via* the modulation of cellular PI3K/Akt and p38 MAPK pathways.

## Introduction

Herpes simplex virus type 1 (HSV-1) is a neurotropic double-stranded DNA virus that belongs to the family of alpha herpesviruses, with humans as their only hosts (Fatahzadeh and Schwartz, 2007). Approximately 50–90% of the world's population is seropositive to HSV-1, making its infection common (Harfouche et al., 2019; Khadr et al., 2019). After the virus enters the host through the mucous membrane or skin, it proceeds to sensory nerve endings and ganglia, where a lifelong latency is established and no cure is possible (Bhutta et al., 2021). Symptoms of HSV-1 infection usually manifest as herpes labialis and skin blisters, but in some cases, the infection can lead to particularly severe conditions, including viral keratitis, viral encephalitis, and even death (Suzich and Cliffe, 2018; Yan et al., 2020). Accumulating evidence points to HSV-1 infection playing a critical role in the development of neurodegenerative diseases, such as Alzheimer's disease (Agostini et al., 2018) and Parkinson's disease (Caggiu et al., 2016). Currently, there is no clinically approved vaccine for preventing HSV-1 infection. FDA-approved therapeutic agents against the disease consist mainly of nucleoside analogs, like acyclovir, penciclovir, and valacyclovir, which target viral DNA polymerase to prevent virus replication (De Clercq, 2011, 2013). However, drug-resistant strains and severe side effects often emerge after long-term use of these antiviral drugs, particularly in immunocompromised patients (Vere Hodge and Field, 2013). Hence, the development of novel potent anti-HSV-1 viral therapies with mechanisms that differ from those of nucleoside analogs is a necessity.

Sophoridine (known as 5β-Matrine, SRI, CAS No.:6882–68–4, Figure 1A) is a naturally occurring quinolizidine alkaloid mainly isolated and identified from the seeds of *Sophora alopecuroides* L. (Liang et al., 2012; Weng et al., 2016). The SRI hydrochloride injection was approved as an anti-cancer agent in China in 2005. Research has demonstrated a wide range of pharmacological activities of SRI, including anti-cancer (Wang et al., 2019), anti-inflammatory (Huang et al., 2014), antiviral (Ren et al., 2019), anti-arrhythmia (Hu et al., 2016), and analgesia (Yan et al., 2014) in recent years. Importantly, there is also growing evidence that the alkaloid has non-specific activity against viral infections, including anti-coxsackievirus B3 (CVB3) (Zhang et al., 2006), anti-respiratory syncytial virus (RSV) (Ma et al., 2002), anti-enterovirus 71 (EV71) (Ren et al., 2019), and hepatitis B virus (HBV) (Chen et al., 2016; Zhang et al., 2018). The antiviral mechanism of SRI may be associated with the regulation of cytokines (IFN-γ and IL-10) and the p38 MAPK signaling pathway (Zhang et al., 2006; Chen et al., 2016). However, its inhibition of HSV-1 infection has not been reported until now. In the present study, we provide the first evidence that SRI has an inhibitory activity against HSV-1 infection *in vitro* and also discuss the underlying mechanism of this action.

**Figure 1:**
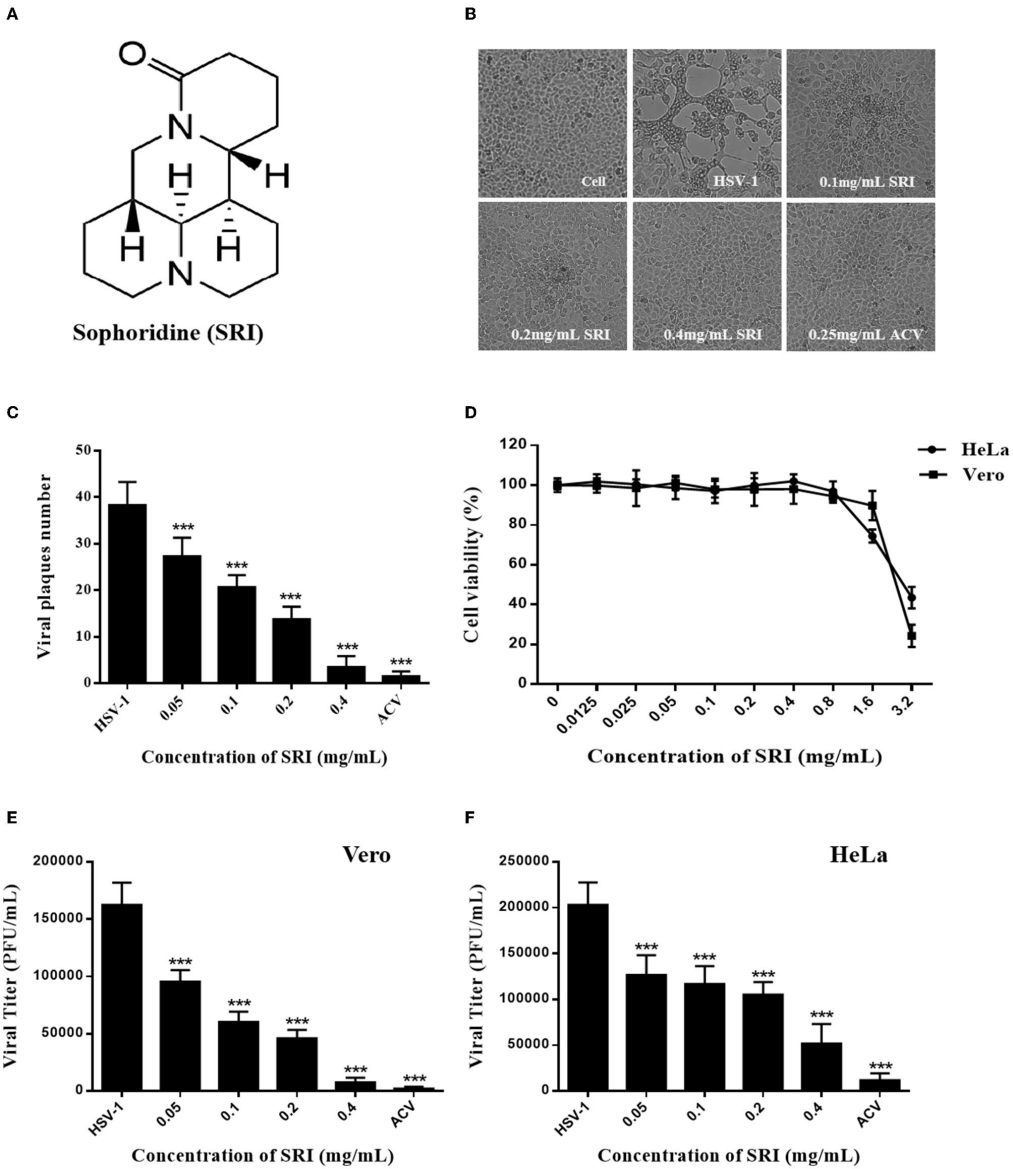
Toxicities and inhibitory effects of SRI on HSV-1 *in vitro*. **(A)** The chemical structure of SRI. **(B)** The cells infected with HSV-1 (MOI = 1) were treated with SRI (0.1, 0.2, and 0.4 mg/mL) or ACV (0.25 mg/mL), and morphological changes of Vero cells were captured under an optical inverted microscope at 72 h post-infection. **(C)** The cells infected with HSV-1 (MOI = 1) were treated with SRI or ACV, and the inhibitory effect of SRI was estimated by plaque assay. The number of plaques on Vero cells infected with HSV-1 was counted. **(D)** Vero and HeLa cells were treated with SRI (from 0.0125 to 3.2 mg/mL) for 48 or 72 h, and cell viability was calculated by MTT assay. **(E,F)** Vero and HeLa cells were infected with HSV-1 (MOI = 1) and were then treated with SRI or ACV for 24 h. The progeny virus was released after three cycles of freezing and thawing of the infected cells, and the progeny virus titer was determined by plaque assay. The results are given as mean ± SD. ^***^p < 0.001 vs. HSV-1 group.

## Materials and Methods

### Compounds, Antibodies, and Reagents

Sophoridine (19113001, purity > 96%) was obtained from Chengdu Must Biotechnology (Chengdu, Sichuan, China) and dissolved to completion to obtain a final concentration of 20 mg/mL in PBS. Acyclovir (1411201) was purchased from Qian Jiang Pharmaceutical (Qianjiang, Hubei, China) and dissolved to completion in PBS (concentration of 10 mg/mL). Antibodies specific for infected cell protein 0 (ICP0, sc-53070), glycoprotein B (gB, sc-56987), and glycoprotein D (gD, sc-21719) were acquired from Santa Cruz Biotechnology (Santa Cruz, CA, USA). Antibodies specific for PI3K (4257), p-PI3K (17366), Akt (4691), p-Akt (4060), mTOR (2983), p-mTOR (5536), p38 (8690), p-p38 (4511), JNK (9252), p-JNK (9251), p-ATF-2(24329), c-jun (9165), and p-c-jun (3270) were procured from Cell Signaling Technology (Beverly, MA, USA). Peroxidase-conjugated goat anti-rabbit and peroxidase-conjugated goat anti-mouse antibodies were obtained from Boster Biological Technology (California, USA). Internal parameters, GAPDH and tubulin, were bought from Servicebio (Wuhan, China).

### Cell Culture and Viral Infection

Vero and HeLa cells were obtained from American Type Culture Collection (ATCC, USA) and cultured in DMEM containing 10% FBS in 5% CO_2_ at 37°C. HSV-1 was purchased from the Wuhan Institute of Virology, Chinese Academy of Sciences. For HSV-1 infection, the virus was diluted in DMEM containing 3% FBS at the indicated multiplicity of infection (MOI) and was added to monolayer cells. HSV-1 was propagated in Vero cells and titrated as previously described (Luo et al., 2020). Briefly, virus reserves were generated by infecting the monolayers of Vero cells for 72 h and then lysing the cells with three freeze-thaw cycles. The resulting cell lysates were separated and stored at −80°C. The HSV-1 titer was measured using the plaque-forming units (PFU) assay.

### Cytotoxicity Assay

The *in vitro* cytotoxicity capability of SRI on Vero and HeLa cells was evaluated using the MTT assay (Biofroxx, Germany), as described earlier with minor modifications (Goswami et al., 2018). Briefly, Vero and HeLa cells placed in 96-well plates were treated with serially diluted SRI (0.0125, 0.025, 0.05, 0.1, 0.2, 0.4, 0.8, 1.6, and 3.2 mg/mL), incubated at 37°C in 5% CO_2_, subjected to the MTT assay after 48 or 72 h following the manufacturer's protocol, and their absorbance was measured at 490 nm using a Varioskan LUX multifunctional microplate reader (Thermo Scientific, Waltham, MA). The half-maximal cytotoxic concentration (CC_50_) of SRI in Vero and HeLa cells was determined, and cell viability was calculated as the percentage of the drug-treated group relative to the untreated control group.

### Plaque Reduction Assay

The plaque reduction assay was conducted following the methods reported in the literature (Li et al., 2019). Briefly, Vero cell monolayers placed in 12-well plates were infected with HSV-1 (MOI = 1.5) at 37°C. Two hours later, the viral inoculum was removed, and 1 mL of methylcellulose overlay (2% methylcellulose, with 2 × final testing concentrations of SRI in a 1:1 ratio) was added to each well and left for incubation at 37°C and 5% CO_2_ for 72 h. The cells were eventually fixed in 4% paraformaldehyde (PFA; Boster Biological Technology, California, USA) and stained with 1% crystal violet (Solarbio, Beijing, China) for plaque counting.

### Progeny HSV-1 Yield Assay

The antiviral activity of SRI was evaluated *via* titration of infectious virions in SRI-treated cells, as described previously (Li et al., 2017, 2018). Briefly, Vero and HeLa cell monolayers placed in 12-well plates were infected with HSV-1 (MOI = 1) for 2 h and treated with SRI (0.05, 0.1, 0.2, and 0.4 mg/mL). The medium was then discarded after 24 h and replaced with 1 ml of fresh medium in each well. Eventually, the cells were frozen and thawed for three cycles to release intracellular progeny virus particles. The progeny virus titers were performed using a plaque-forming assay. The half-maximal effective concentration (EC_50_) of SRI was estimated.

### Cytopathic Effect Inhibition Assay

The anti-HSV-1 effect of SRI was scrutinized using the cytopathic effect (CPE) inhibition assay (Wang et al., 2011; Chu et al., 2020). Briefly, Vero cell monolayers placed in 96-well plates were infected with HSV-1 (MOI = 1) for 2 h, incubated with serial concentrations of SRI (0.05, 0.1, 0.2, and 0.4 mg/mL) after removal of the viral inoculum, and the cytopathic effects of SRI were observed and photographed after 72 h.

### Pre-Treatment of Virus, Anti-Attachment, and Anti-Penetration Assays

The assay was conducted as previously described, with minor modifications (Shen et al., 2019; Wang et al., 2020).

(a) Pre-treatment of the virus: HSV-1 was pretreated with various concentrations of SRI (0.05, 0.1, 0.2, and 0.4 mg/mL) and incubated at 37°C for 1 h before infection. The viral inoculum was added to Vero monolayer cells at 37°C and discarded after 2 h. Subsequently, the infected cells were washed with PBS two times and subjected to the plaque reduction assay for counting the number of plaques.

(b) Anti-attachment assay: Vero monolayer cells were pre-chilled at 4°C for 1 h, after which the medium was replaced with HSV-1 and various SRI concentrations (0.05, 0.1, 0.2, and 0.4 mg/mL) and incubated at 4°C to allow binding but not cellular uptake. Following the 2 h of incubation, the cells were washed twice with cold PBS to remove the unbound virus, and the number of plaques was counted using the reduction assay.

(c) Anti-penetration assay: Vero monolayer cells were pre-chilled at 4°C for 1 h, after which HSV-1 was added to their milieu for incubation at 4°C for 2 h. Subsequently, the viruses were removed, Vero cells were incubated with various SRI concentrations (0.05, 0.1, 0.2, and 0.4 mg/mL) at 37°C for 2 h to facilitate viral penetration, the cells were washed twice with PBS, and plaque numbers were counted using the reduction assay.

### Western Blot Assay

Cells were infected with HSV-1, incubated with SRI (0.1, 0.2, or 0.4 mg/mL) or ACV (0.25 mg/mL), lysed by adding the RIPA lysis buffer (Beyotime, Shanghai, China) on ice for 30 min, and centrifuged at 4°C for 10 min at 12,000 × g. Total protein concentrations in the supernatants were measured with the BCA Protein Assay Kit (Beyotime, Shanghai, China), and the protein samples were adjusted to the same concentrations. Samples were then separated on 10% sodium dodecyl sulfate-polyacrylamide gel electrophoresis (SDS-PAGE) and transferred onto pre-equilibrated PVDF membranes. The membranes were sealed in 5% BSA for 1.5 h, co-incubated with the primary antibody or anti-tubulin and GAPDH antibodies as control at 4°C overnight, co-incubated with a secondary antibody at room temperature for 2 h, and their contents were visualized using an ECL Western Blot Detection Kit (Beijing 4A Biotech Co., Ltd, Beijing, China).

### Real-Time PCR

Cells were infected with HSV-1 (MOI = 10) and treated with SRI (0.1, 0.2, and 0.4 mg/mL) or ACV (0.25 mg/mL). Total RNA from the cell samples was extracted using the Cell Total RNA Isolation Kit (Chengdu Fuji Biotechnology, Chengdu, China) following the manufacturer's protocol. cDNA was synthesized in a reverse transcriptase reaction using FastKing gDNA Dispelling RT SuperMix (Tiangen Biotech, Beijing, China). Relative mRNA expression was measured using SuperReal PreMix Plus SYBR Green (Tiangen Biotech, Beijing, China) on a CFX Connect™ Real-Time PCR Detection System. Primer pairs used in this study are listed in Table 1. Gene transcription levels were standardized to GAPDH mRNA. Analysis findings were calculated based on the 2^−ΔΔCT^ threshold cycle method.

**Table 1:** Primers used for RT-qPCR in this study.

**Genes**	**Forward primer sequence**	**Reverse primer sequence**
HSV-1 ICP0	F: 5′-TGTGCACGGATGAGATCG-3′	R: 5′-TCGTTCACGATCGGGATG-3′
HSV-1 ICP8	F: 5′-ATGGACAAGGTAACCATCGG-3′	R: 5′-TTGAAAAACGGAAGGGGGTA-3′
HSV-1 ICP22	F: 5′-CGCCGCAGAAGACCGCAAGT-3′	R: 5′-TGTCGCTGCACGGATAGGG-3′
HSV-1 US6	F: 5′-GTCATGGAGTACACCGAATGCT-3′	R: 5′-TCTTCACGAGCCGCAGGTAC-3′
HSV-1 TK	F: 5′-CGATGACTTACTGGCGGGTGT-3′	R: 5′-GCGTCGGTCACGGCATAA-3′
HSV-1 UL27	F: 5′-GCCTTCTTCGCCTTTCGC-3′	R: 5′-GCCATGTACCGTATCATCTCCC-3′
GAPDH	F: 5′-CAGCCTCAAGATCATCAGCAA-3′	R: 5′-CCATCACGCCACAGTTTCC-3′

### Statistical Analysis

Statistical significance was analyzed using the SPSS 21.0 software. Data are expressed as the mean ± standard deviation (SD) values from at least three experiments. All data analyses were conducted using one-way ANOVA or the *t*-test. Statistical significance was set at *P* < 0.05.

## Results

### Cytotoxicity and Anti-HSV-1 Activity of SRI

Sophoridine is a widely distributed natural quinolizidine alkaloid with various pharmacological properties, including anti-cancer, antiviral, anti-inflammatory, and anti-arrhythmia. The structure of this compound is shown in Figure 1A. Surprisingly, we observed that SRI significantly inhibited the formation of the HSV-1-induced CPE in Vero monolayers (Figure 1B). Furthermore, the antiviral activity of SRI estimated using a plaque reduction assay showed that SRI markedly subdued the number of viral plaques in a dose-dependent manner (Figure 1C). The MTT assay-determined cytotoxicity of SRI on the two cell lines used demonstrated its low cytotoxicity on Vero and HeLa cells, with (SI = CC50/EC50). values of 2.649 and 3.099 mg/mL, respectively (Figure 1D).

Infectious HSV-1 particles were released through three cycles of freezing and thawing of the infected Vero and HeLa cells, and progeny viral infectivity was titrated using the plaque reduction assay. As shown in Figures 1E,F, SRI drastically reduced the viral titer of HSV-1 progeny virus in Vero and HeLa cells, with the EC_50_ values of 0.068 and 0.137 mg/mL, respectively. Hence, the selection indexes (SI = CC_50_/EC_50_) of SRI in the two infected cells were 38.96 and 22.62, respectively, indicating that SRI is a less cytotoxic but highly efficient drug. Taken together, these results suggest that SRI exhibited considerable anti-HSV-1 activity in Vero and HeLa cells.

### Effects of Different SRI Treatment Conditions on HSV-1 Infection

**Figure 2:**
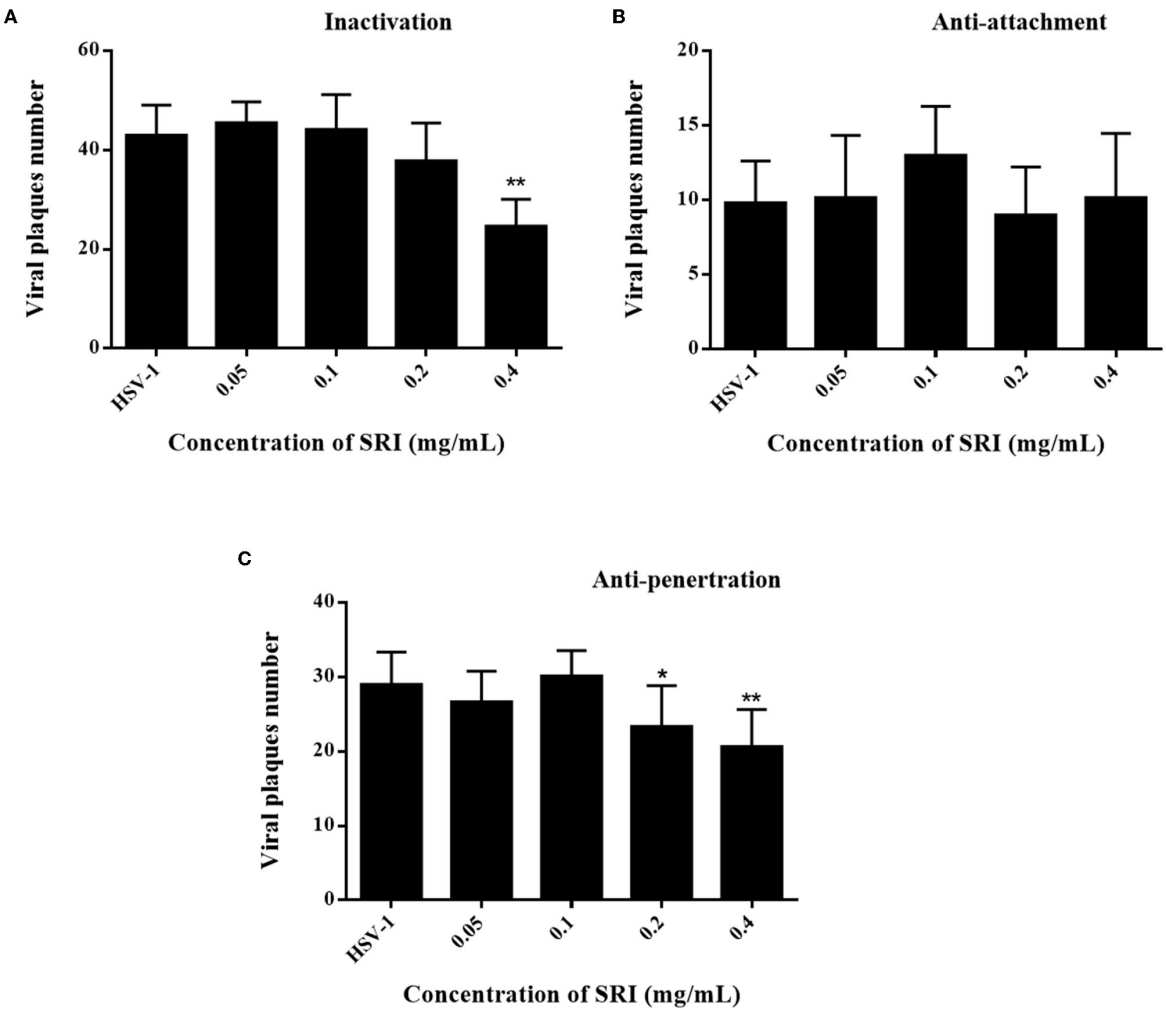
Effects of different treatment conditions of SRI on HSV-1 infection. **(A)** Pre-treatment of virus: HSV-1 was pre-treated with SRI (0.05, 0.1, 0.2, and 0.4 mg/mL) and incubated at 37°C for 1 h. The viral inoculum was added to a Vero cell monolayer and incubated for 2 h. Then, a plaque reduction assay was performed to calculate the number of plaques. **(B)** Viral attachment: pre-chilled monolayer of Vero cells was infected with HVS-1 and SRI (0.05, 0.1, 0.2, and 0.4 mg/mL) and incubated for 2 h at 4°C to allow binding (but not cellular uptake). Then, a plaque reduction assay was performed to calculate the number of plaques. **(C)** Viral penetration: HSV-1 was added to a pre-chilled monolayer of Vero cells. After incubation at 4°C for 2 h, the viruses were removed and Vero cells were incubated with SRI (0.05, 0.1, 0.2, and 0.4 mg/mL) at 37°C for 2 h to facilitate viral penetration. Then plaque reduction assay was performed to calculate the number of plaques. The results are presented as mean ± SD. ^*^p < 0.05, ^**^p < 0.01 vs. HSV-1 group.

Administration of SRI after HSV-1 viral infection suppressed the replication of HSV-1 and diminished the viral titers of the progeny virus (Figures 1D,E), indicating that SRI inhibited HSV-1 infection. In addition, we also examined the ability of SRI to target other stages of HSV-1 infection, including preconditioning viruses, attachment, and penetration. Our data revealed that pretreating HSV-1 with SRI for 1 h before infection significantly reduced viral plaque formation compared to the non-treated viral control group (Figure 2A). However, incubation of HSV-1 with SRI during the viral-cell attachment phase scarcely decreased viral plaque formation *in vitro* (Figure 2B). SRI can inhibit the formation of viral plaques during viral penetration (Figure 2C). Administering SRI during the replication phase after the entry of the virus into the cells substantially repressed viral plaque formation (Figure 1C). Most importantly, the plaque inhibition rates using 0.4 mg/mL SRI for direct inactivation, penetration, and replication of the virus were 42.6%, 28.7%, and 90.2%, respectively. These findings suggested that SRI may directly inactivate viral particles and interfere with some steps of the HSV-1 life cycle after adsorption, particularly during the replication phase.

### SRI Inhibits the Expression of HSV-1 Immediate-Early, Early, and Late Genes

**Figure 3:**
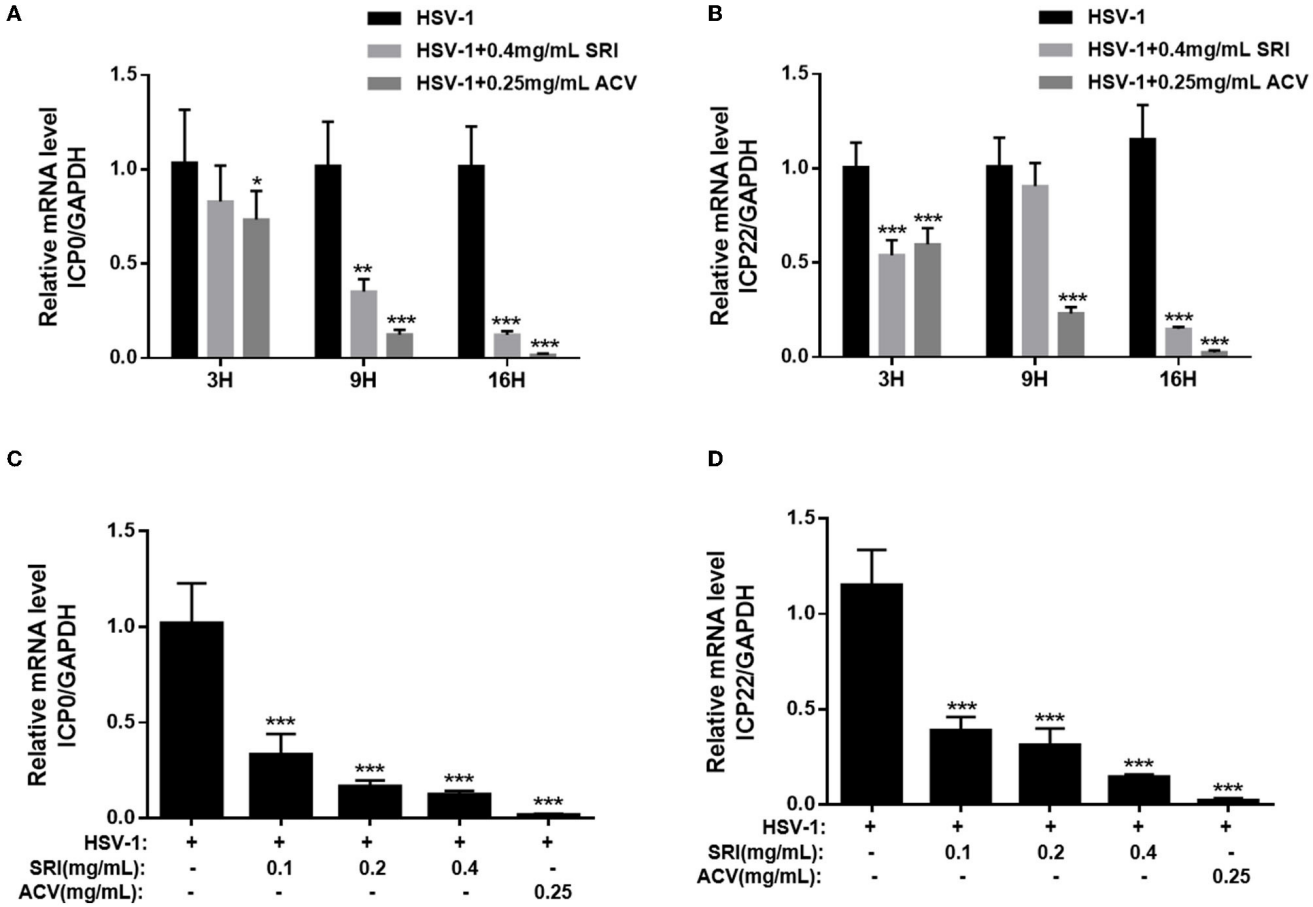
SRI repressed HSV-1 IE gene expression. **(A–D)** Confluent cells were infected with HSV-1 for 2 h and then treated with the designated concentrations of SRI (0.05, 0.1, 0.2, and 0.4 mg/mL) or ACV (0.25 mg/mL). Total RNA was extracted at the indicated time points (3, 9, and 16 h) and RT-PCR was employed to analyze the ICP0 and ICP22 mRNA levels **(A,B)**. The effects of SRI and ACV on ICP0 and ICP22 genes of the virus were determined after 16 h **(C,D)**. The level of gene transcription was normalized by GAPDH. The data are presented as mean ± SD. ^*^p < 0.05, ^**^p < 0.01, and ^***^p < 0.001, vs. HSV-1 group.

Because previous investigations showed the drug effect to be most notable during the HSV-1 replication phase (Figure 1C), we explored the ability of SRI to inhibit the virus at this phase in further experiments. HSV-1 infection involves the regulated expression of three phases of viral genes: immediate-early (IE**)**, early (E), and late (L) genes in that order (Su et al., 2017; Dremel and DeLuca, 2019). The IE genes, expressed first after virus entry and capsid decomposition, regulate the expression of E and L viral genes. Hence, we initially scrutinized the time course of the inhibitory effect of SRI on one of the IE genes, infected cell polypeptide 0 (ICP0), within 16 h of HSV-1 infection *via* RT-PCR. The results showed that SRI reduced ICP0 expression at 3, 9, and 16 h (Figure 3A), and the expression of ICP0 was inhibited in a dose-dependent manner at 16 h (Figure 3C). Meanwhile, we also examined the time course of the inhibitory effect of SRI on the ICP22 gene, which along with ICP0 is the most important IE gene, expression during the early stages of HSV-1 infection. The results revealed that SRI also harbored an inhibitory activity on ICP22 expression (Figures 3B,D). The time course of SRI to show its effect on ICP22 gene was similar to the time course required for the ICP0 gene (Figure 3A). The above-mentioned results showed that SRI can inhibit HSV-1 replication *via* interfering with HSV-1 IE gene expression.

**Figure 4:**
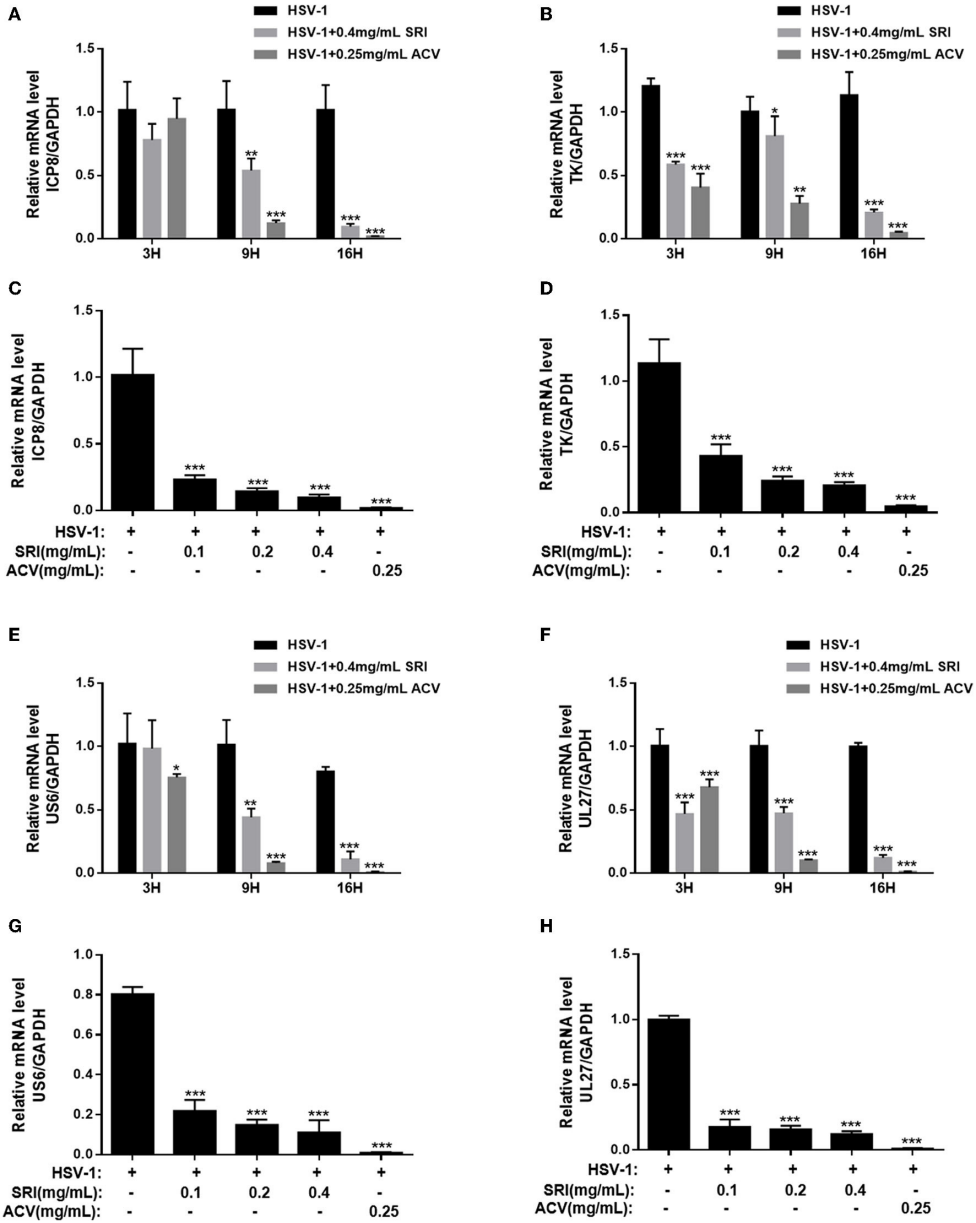
Effect of SRI on HSV-1 E and L gene expression. (**A–H**) Confluent cells were infected with HSV-1 for 2 h and then treated with the designated concentrations of SRI (0.05, 0.1, 0.2, and 0.4 mg/mL) or ACV (0.25 mg/mL). Total RNA was extracted at the indicated time points (3, 9, and 16 h) and RT-PCR was employed to analyze the ICP8 **(A)**, TK **(B)**, US6 **(E)**, and UL27 **(F)** mRNA levels. The effects of SRI and ACV on ICP8, TK, US6, and UL27 genes of virus were determined after 16 h **(C,D,G,H)**. The level of gene transcription was normalized by GAPDH. The data were presented as mean ± SD. ^*^p < 0.05, ^**^p < 0.01, and ^***^p < 0.001, vs. HSV-1 group.

The ability of SRI to suppress the HSV-1 E gene expression was also investigated. ICP8, a single-strand DNA-binding protein that acts as an E protein, is very crucial in the late gene expression of the virus (Darwish et al., 2015; Weerasooriya et al., 2019). As shown in Figures 4A,C, SRI blocked the expression of the ICP8 virus gene. Similarly, it inhibited the expression of another early gene, TK (Figures 4B,D). The suppressive activity of SRI on the E gene was consistent with the results on the IE gene expression. To further understand the antiviral activity of SRI against HSV-1 replication, we monitored the decrease in gD and gB levels, which are HSV-1 membrane proteins representing late gene products in the viral life cycle. As shown in Figures 4E,G, RT-PCR analysis found that SRI effectively inhibited the gD (US6) mRNA transcript of HSV-1 in a dose-dependent manner, which was consistent with the decrease of E gene expression. The expression of another late gene product, gB (UL27), a highly conserved glycoprotein and presumably directly involved in the fusion of viruses with host cell membranes (Rogalin and Heldwein, 2016), was also researched, and its results were consistent with the expression of gD (Figures 4F,H). These results indicated that SRI obstructs the replication of the HSV-1 by suppressing the expression of viral late genes.

### SRI Inhibited the Protein Expression of HSV-1

**Figure 5:**
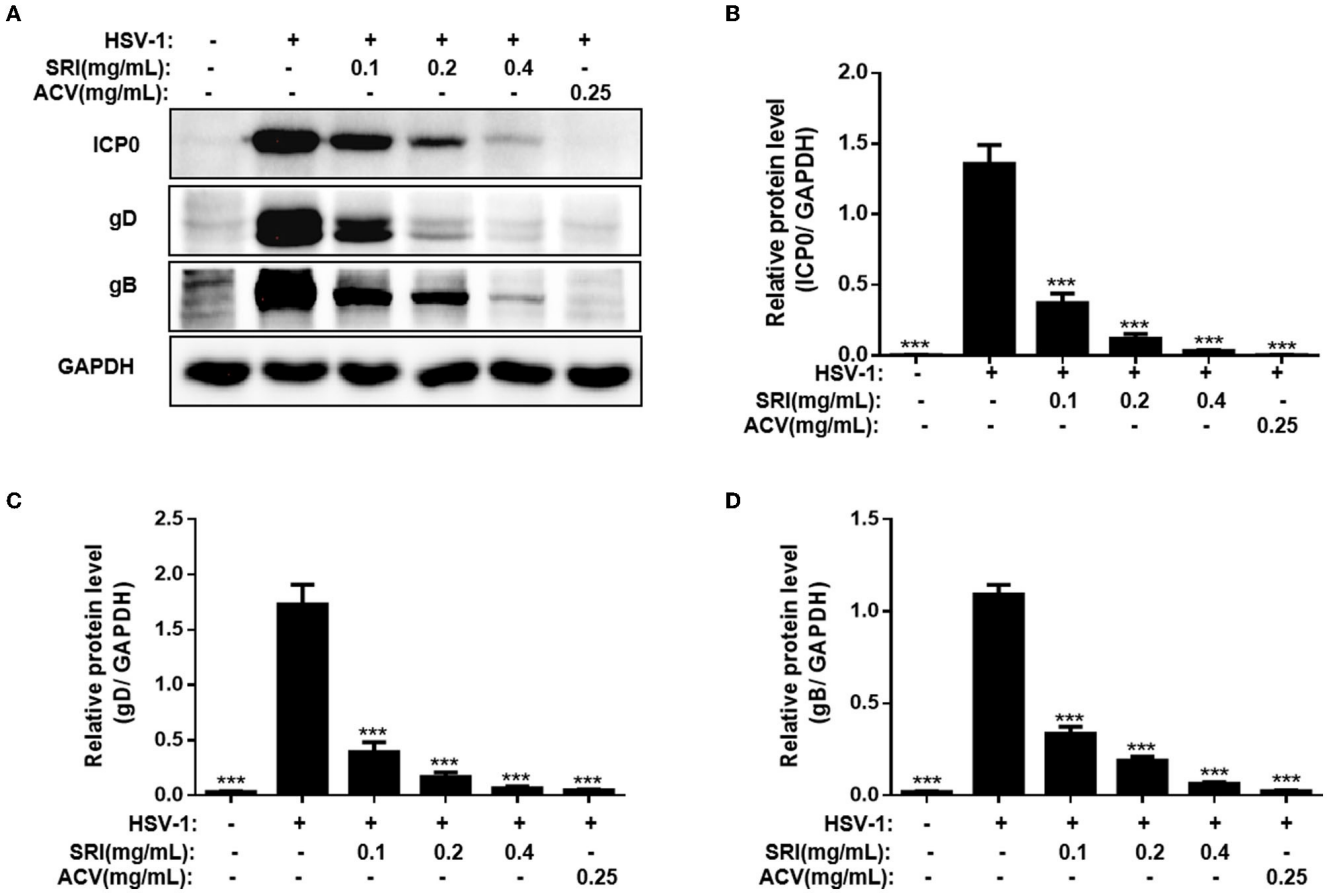
SRI inhibited the HSV-1 ICP0, gB, and gD protein expression levels. **(A–D)** Cells grown in six-well plate were infected with HSV-1 and treated with SRI (0.05, 0.1, 0.2, and 0.4 mg/mL) or ACV (0.25 mg/mL). At 24 h post-infection, the cellular proteins were harvested and determined using Western blot analysis using primary antibodies to HSV-1 ICP0, gB, gD, and GAPDH. GAPDH was used as a standard loading control. The quantification results of the Western blot analysis of ICP0 **(B)**, gD **(C)**, and gB **(D)**. The data are expressed as mean ± SD. ^***^p < 0.001, vs. HSV-1 group.

The ability of SRI to hinder the transcription of viral genes has been demonstrated in our previous results. To further determine whether SRI suppresses HSV-1 protein expression, we evaluated the impact of SRI on the viral protein levels (ICP0, gB, and gD) using Western blot analysis. gB and gD proteins are essential for the entry of HSV-1 into the host cells, and the ICP0 protein of HSV-1 is one of the first proteins expressed during the replication cycle of HSV-1. As shown in Figures 5A–D, Western blot analysis showed the efficiency of SRI in blocking the expression of viral proteins ICP0, gB, and gD. Similarly, the inhibitory effect of SRI was comparable to the expression of the genes. These results indicate that SRI inhibits HSV-1 replication by preventing the gene and protein expression of the virus.

### SRI Inhibited HSV-1 Replication by Blocking PI3K/Akt Pathway

**Figure 6:**
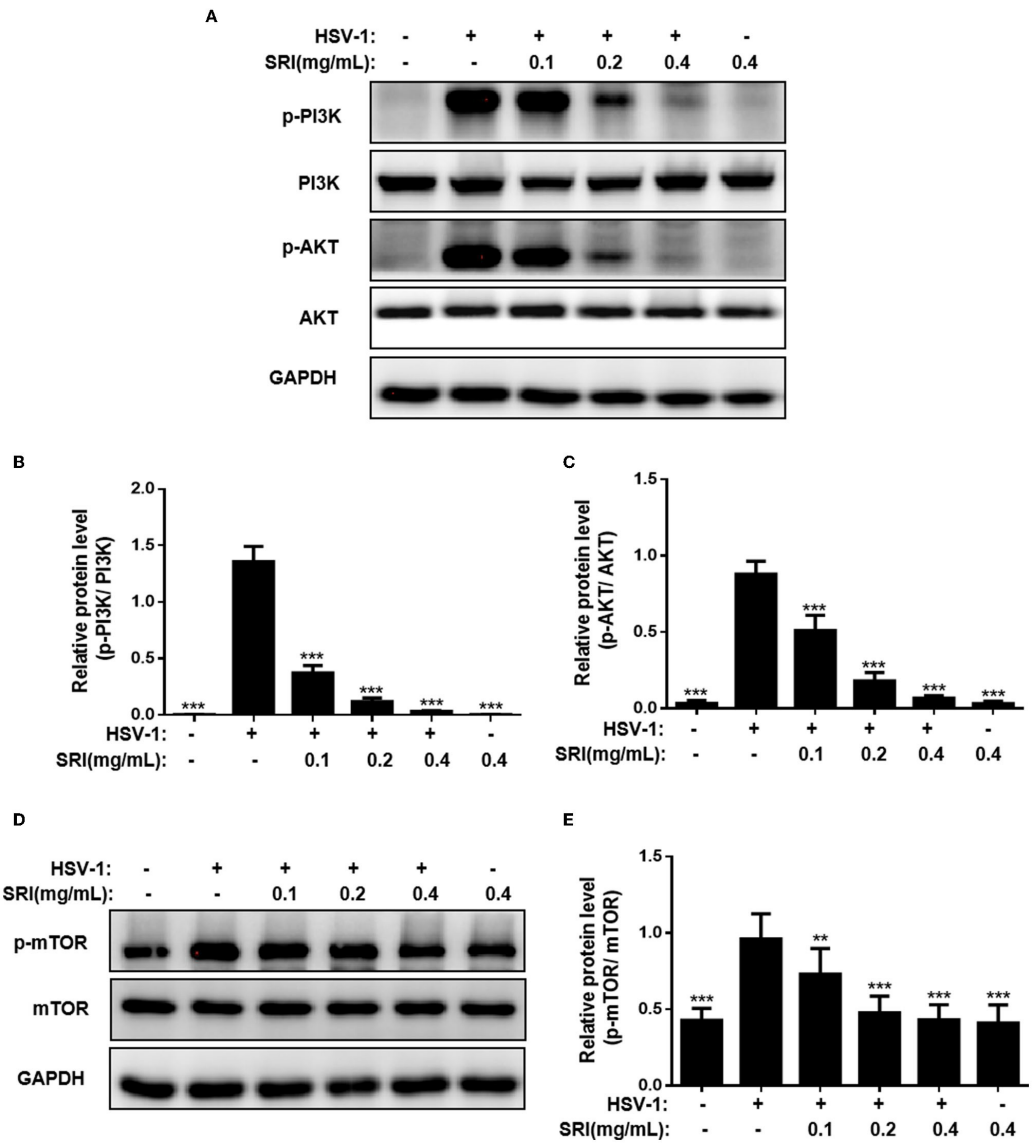
SRI inhibited HSV-1 replication through blocking of PI3K/Akt pathway. **(A–E)** The infected cells of HSV-1 (MOI = 1) were treated with or without SRI (0.1, 0.2, and 0.4 mg/mL), and the protein levels of p-PI3K, PI3K, p-Akt, and Akt were evaluated using Western blotting analysis. Blots were also probed for GAPDH, which was used as a loading control. Plots quantifying the immunoblots as ratios for p-PI3K/PI3K **(B)**, p-AKT/Akt **(C)**, and p-mTOR/mTOR **(E)**, respectively. The results shown are representative of three independent experiments. The results are presented as mean ± SD values. ^**^p < 0.01 and ^***^p < 0.001 vs HSV-1 group; ns, not significant.

In our research study, SRI repressed the replication of HSV-1; therefore, we used the Western blot analysis to assess the ability of SRI to impact HSV-1 infection-related signaling pathways to produce this effect. The PI3K/Akt signaling pathway was supposed to be essential for viral replication, and the inhibitors of the PI3K/Akt signaling pathway allegedly inhibit both HSV-1 entry and fusion (Eaton et al., 2014; Li et al., 2020). First, we examined whether HSV-1 infection could activate the PI3K/Akt signaling pathway. The activation of cellular PI3K and Akt proteins was markedly higher in the HSV-1-infected control group than in the normal control group (Figure 6A), indicating that HSV-1 infection and replication stimulate the activation of the PI3K/Akt pathway. Treatment with SRI drastically reduced the degree of PI3K phosphorylation in a dose-dependent manner compared to the HSV-1-infected control group (Figure 6B). Moreover, the presence of SRI also substantially diminished the level of phosphorylated Akt (Figure 6C). Herein, we looked into the possibility of SRI affecting the expression of PI3K and Akt proteins in the uninfected cells. We found that SRI treatment did not trigger the activation of the PI3K/Akt pathway in the uninfected cells (Figures 6A–C). Additionally, our exploration of the downstream target of the PI3K/Akt pathway revealed that sophoridine impeded mTOR protein phosphorylation in a dose-dependent manner (Figures 6D,E). In conclusion, these outcomes point to SRI being able to inhibit HSV-1-induced PI3K/Akt activation, thereby preventing HSV-1 replication.

### SRI Inhibited HSV-1 Replication by Blocking p38 MAPK Activation but Showed Little Effect on JNK Activation

**Figure 7:**
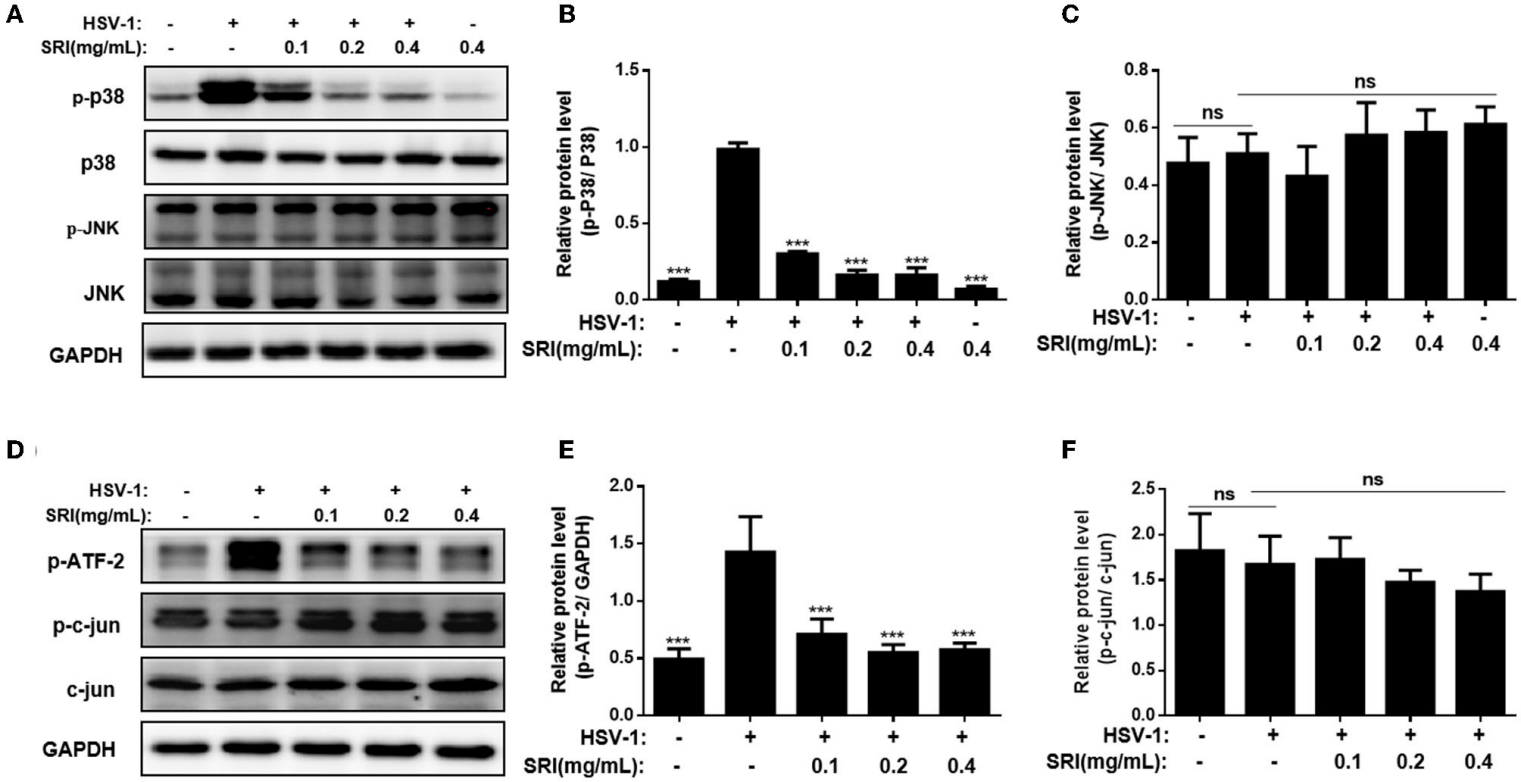
SRI restrained HSV-1 replication through blocking of P38 MAPK pathway. **(A,D)** The infected cells of HSV-1 (MOI = 1) were treated without or with SRI (0.1, 0.2, and 0.4 mg/mL), and then the protein levels of p-p38, p38, JNK, p-JNK, p-ATF-2, p-c-jun, and c-jun were evaluated through Western blot analysis. Blots were also probed for GAPDH and tubulin proteins as loading controls. **(B,C,E,F)** Plots quantifying the immunoblots as ratios for p-P38/P38 **(B)**, p-JNK/JNK **(C)**, p-ATF-2/GAPDH **(E)**, and p-c-jun/c-jun **(F)**, respectively. The results were presented as mean ± SD. ^***^p < 0.001, vs. HSV-1 group; ns, not significant.

To further understand the action mechanism of SRI in the cells after HSV-1 infection, the impact of SRI on the HSV-1-mediated JNK/p38 signaling pathway was examined. The JNK and p38 signaling pathways are the two main pathways involved in the stress activation and inflammatory response induced by various viral infections (Yang et al., 2016; Cheng et al., 2020). In the past, researchers have demonstrated that an HSV-1 infection activates the JNK and p38 MAPK pathways, and plays an essential role in HSV-1 replication (Kopecky-Bromberg et al., 2006; Chen et al., 2015; Wan et al., 2021). As shown in Figures 7A–C, the phosphorylation of p38 in the HSV-1 infection control group was markedly elevated compared to the normal control group (Figure 7B); however, JNK phosphorylation in the Vero cells was not affected (Figure 7C), suggesting that the infection only activated the p38 MAPK pathway. Moreover, SRI treatment significantly decreased the HSV-1-mediated phosphorylation levels of p38 MAPK in Vero cells. These results demonstrated that the antiviral activity of SRI may be linked to the inhibition of the p38 MAPK signaling pathway.

Determining whether HSV-1 infection affects the phosphorylation of c-jun and ATF-2, downstream targets of the JNK and p38 MAPK pathways, was also important (Sloan and Jerome, 2007; Su et al., 2017). The phosphorylation of ATF-2 considerably increased after HSV-1 infection; however, SRI treatment markedly subdued this reaction in a dose-dependent manner (Figures 7D,E). The results of Western blot analysis showed that SRI had no obvious impact on c-jun and its phosphorylation level (Figures 7D,F) and no notable influence on the JNK signaling pathway in the HSV-1 infection model in Vero cells. In summary, these findings indicate that SRI interferes with HSV-1 replication in a manner possibly associated with the suppression of the p38 MAPK pathway, rather than the activation of the JNK pathway.

## Discussion

Sophoridine is among the quinolizidine alkaloids that have been reported to exhibit noticeable antiviral activity against various viruses including CVB3 (Zhang et al., 2006), RSV (Ma et al., 2002), HBV (Chen et al., 2016; Zhang et al., 2018), and EV71 (Ren et al., 2019). In this study, we highlight the antiviral activities of SRI against HSV-1 replication in two cell types, with the virus exhibiting very low toxicity. SRI displayed significant antiviral activity by inhibiting viral plaque formation and progeny virus production. Meanwhile, we also assessed the impact of SRI on HSV-1 infection under different treatment conditions, including preconditioning viruses, attachment, and penetration. The findings of viral inactivation, penetration, and replication assays suggest that SRI directly inactivates virus particles and inhibits virus penetration. Interestingly, incubating HSV-1 with SRI during the viral-cell attachment phase scarcely diminished viral plaque formation. In a nutshell, we found that SRI can inactivate viral particles directly and block several steps in the HSV-1 life cycle after adsorption, particularly during viral replication.

Upon entering the host cell, the virus uses proteins and nucleic acids present in the host cell nucleus for active replication. Transcriptional regulation of HSV-1 occurs at three successive stages of gene expression, including immediate-early (IE), early (E), and late (L) genes (Li et al., 2018; Dremel and DeLuca, 2019). The IE gene is expressed first after the virus entry and capsid decomposition and regulates the expression of E and L viral genes. Our further exploration of the effect of SRI on replication-related genes showed that SRI markedly inhibited the expression of viral IE genes (ICP0 and ICP22), early (ICP8 and TK) genes, and late (gB and gD) genes, with the protein results confirming that it can hinder the expression of replication-related proteins. However, the mechanism underlying the suppression of HSV-1 replication by SRI remains unclear.

The PI3K/Akt is a critical signaling pathway that regulates many essential biological activities (Pompura and Dominguez-Villar, 2018). Research has highlighted the ability of a virus to enhance its replication, transcription, and translation functions by regulating the PI3K/Akt signaling pathway. The PI3K/Akt signaling pathway can also promote the entrance, replication, delay, and activation of the virus (Diehl and Schaal, 2013; Chen et al., 2017; Zhan et al., 2020). Reports in the past have equally shown that the modulation of PI3K/Akt signaling pathways is critical to HSV-1 replication in the host cells (Eaton et al., 2014; Ke et al., 2019; Li et al., 2020). Since SRI supposedly inhibits the PI3K/Akt signaling pathway induced by various pathological factors, we examined whether its anti-HSV-1 activity is linked to the inhibition of the pathway (Wang et al., 2017), and the results revealed that SRI treatment considerably repressed the HSV-1 infection-induced phosphorylation of PI3K, Akt, and mTOR, consistent with a previous study (Li et al., 2020). Considering that efficient replication of HSV-1 requires the PI3K/Akt pathway (Tiwari and Shukla, 2010), we speculated that sophoridine interferes with the activation of the PI3K/Akt pathway, thus further inhibiting HSV-1 replication.

c-Jun N-terminal kinase (JNK) and p38 MAPK kinase are two major members of the MAPK family that activate various stimuli, including pro-inflammatory cytokines, osmotic shock, genotoxic factors, and bacteria (Kyriakis and Avruch, 2001). Activated JNK and p38 MAPK can transmit upstream signals to downstream factors and mediate a series of cellular responses, such as apoptosis, differentiation, growth, and immune response (Wagner and Nebreda, 2009; Sui et al., 2014). Both kinases are stimulated by several viruses or virus-related proteins, including hepatitis B/C virus (HBV/HCV) (Yang et al., 2019; Cheng et al., 2020), HIV-1 (Swepson et al., 2016), CVB3 (Zheng et al., 2019), HSV-1 (Chen et al., 2015; Wan et al., 2021), varicella-zoster virus (Liu et al., 2012) (VZV), Barr virus (EBV) (Yang et al., 2016; Li et al., 2021), and severe acute respiratory syndrome (SARS) coronavirus (Kopecky-Bromberg et al., 2006), suggesting that the p38 MAPK and JNK pathways are pivotal to virus-infected cell survival and virus replication. Mounting data show that SRI can suppress the activation of JNK/p38 induced by a variety of pathological factors (Chen et al., 2016; Liu et al., 2016; Xu et al., 2017). Therefore, we postulate that the JNK/P38 signaling pathway possibly participates in the antiviral activity of SRI.

Our data revealed that HSV-1 infection leads to the strong phosphorylation of the p38 protein, which is markedly alleviated by SRI treatment in a dose-dependent manner. Although HSV-1 induced a slight increase in the phosphorylation of JNK, SRI did not significantly inhibit JNK phosphorylation in HSV-1-infected cells. Additionally, c-jun and ATF-2 are two components of the transcription factor AP-1, which regulates the gene expression in response to various stimuli, such as growth factors, cytokines, stress, and microorganisms (Hess et al., 2004; Song et al., 2014), and are located downstream of JNK and p38. The transcriptional activity of c-jun is controlled by JNK phosphorylation, and ATF-2 is a possible target of the JNK or p38 signaling pathways (Hsieh et al., 2014; Su et al., 2017; Shu-chen Cheng et al., 2019). We found that SRI inhibited HSV-1-induced phosphorylation of ATF-2 but had little impact on c-jun. Based on the observation that SRI inhibits HSV-1-induced phosphorylation of p38 and downstream ATF-2, we speculated that SRI probably acts on the downstream AP-1 of the p38 signaling pathway to influence viral replication; however, this speculation requires further examination. Our findings indicate that SRI displayed potential antiviral activity by modulating the p38 MAPK signaling pathway.

**Figure 8:**
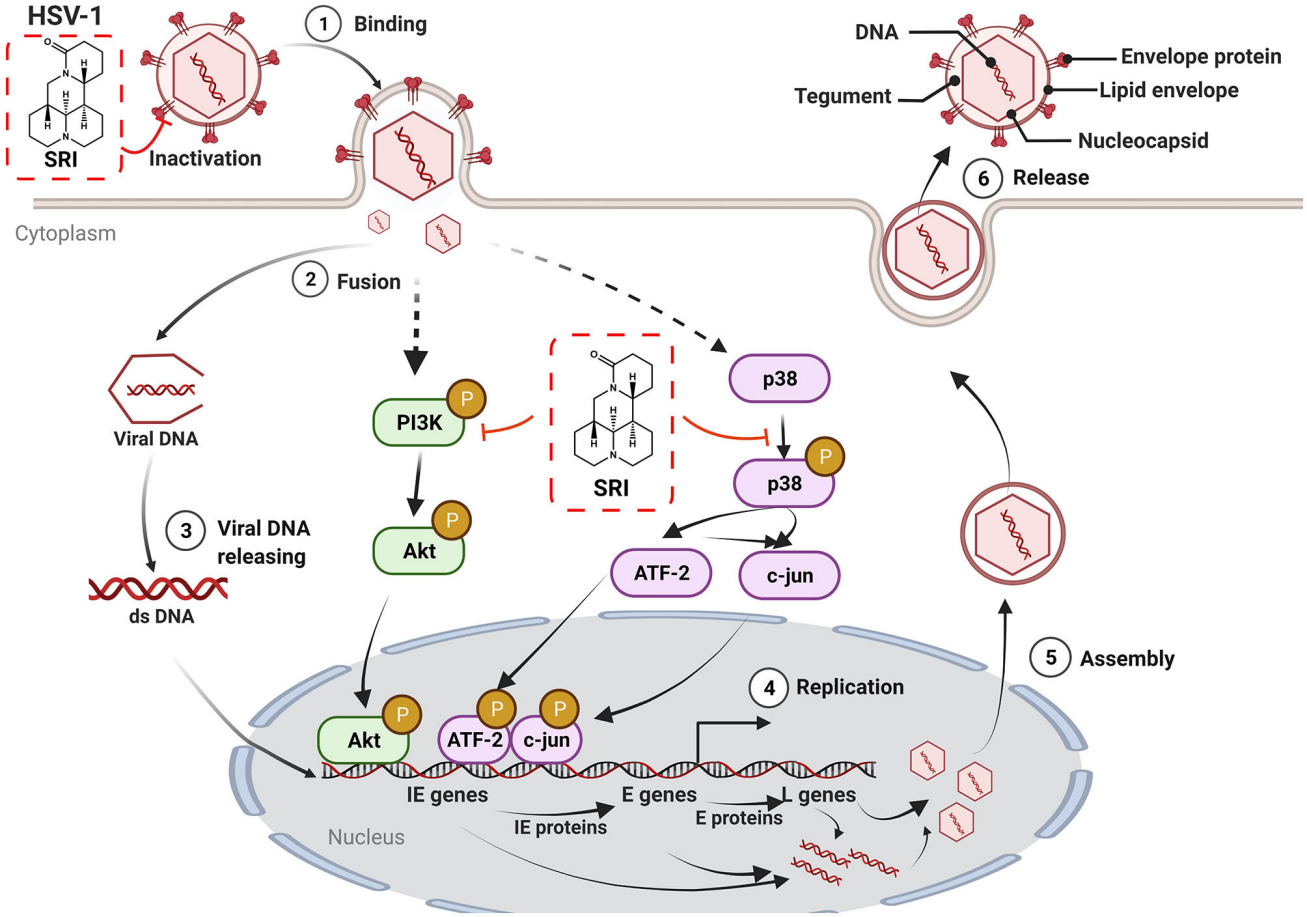
The possible mechanism of action of SRI in HSV-1-infected cells. SRI can directly inactivate HSV-1 virus particles. More importantly, SRI may also suppress the activation of HSV-1-induced cellular PI3K/Akt and p38 MAPK pathways to reduce the subsequent replication of the virus and hence the production of virus progeny particles. Binding (1), fusion (2), viral DNA release (3), DNA replication (4), assembly of virus progeny particles (5), and release (6) (red T line represents inhibition and black arrow marks promotion, respectively).

In summary, we first demonstrated the potent antiviral property of SRI against HSV-1 infection through the direct inactivation of viral particles and blocking of certain steps in the life cycle of HSV-1 after adsorption. Moreover, SRI also significantly inhibited HSV-1-induced activation of PI3K/Akt and p38 MAPK signal pathways, thus preventing HSV-1 replication (Figure 8). Considering that the potential antiviral mechanism of SRI differs from that of other nucleoside analogs, SRI is a promising candidate as a therapeutic agent for drug-resistant HSV-1 infection and deserves further developmental scrutiny. Future research should focus on the molecular mechanisms of SRI *in vivo* with regard to anti-HSV-1 activity and its effect on clinical HSV-1 strains, particularly acyclic virus-resistant strains, which require additional investigation and will contribute to drug development. In a nutshell, this study provides experimental evidence for the utilization of SRI as a potential treatment for HSV-1 infection and related diseases.

## Data Availability Statement

The original contributions presented in the study are included in the article/supplementary material, further inquiries can be directed to the corresponding author/s.

## Author Contributions

QT and FL conceived and designed the study. QT, YL, AY, and BW contributed to carrying out the experiments. QT, FL, AY, JS, and ZR contributed to the data analysis. QT, FL, and YL wrote the manuscript. NZ and YL supervised the research. All authors read and approved the final version of the manuscript.

## Funding

This study was supported by the National Natural Science Foundation of China (82074094), the Xinglin Scholar Research Promotion Project of Chengdu University of Traditional Chinese Medicine (CDTD2018014), the Open Research Fund of Chengdu University of Traditional Chinese Medicine Key Laboratory of Systematic Research of Distinctive Chinese Medicine Resources in Southwest China (2020XSGG002), the Applied Basic Research Project of Science and Technology Department of Sichuan Province (20YYJC0640), and the Key Project of the Education Department of Sichuan Province (18ZB0152 and 18ZA0162).

## Conflict of Interest

The authors declare that the research was conducted in the absence of any commercial or financial relationships that could be construed as a potential conflict of interest.

## Publisher's Note

All claims expressed in this article are solely those of the authors and do not necessarily represent those of their affiliated organizations, or those of the publisher, the editors and the reviewers. Any product that may be evaluated in this article, or claim that may be made by its manufacturer, is not guaranteed or endorsed by the publisher.
